# Supplementary material for: Dangerous appetite: The impact of Trypanosoma cruzi infection on the feeding and defecation behaviors of Triatoma dimidiata sensu lato (Latreille, 1811)
Source: PLoS Negl Trop Dis. 2025 Dec 26;19(12):e0013872. doi: 10.1371/journal.pntd.0013872 (PMC12758822; doi:10.1371/journal.pntd.0013872)
Supplement: Attachment — Submitted filename: 2025-PLoS T. dimidiata.doc [file pntd.0013872.s002.doc]

Comments to the manuscript “Dangerous appetite: The impact of *Trypanosoma cruzi* infection on the feeding and defecation behaviors of *Triatoma dimidiata*”

This is an interesting manuscript from the group of young researchers at UADY linked to the French IRPD group of Dr. Waleckx. As the authors state, this is another of several studies that attempt to clarify whether *T. cruzi* infection sufficiently modifies the behavior of a triatomine species (*T. dimidiata* in this case) to increase the chances of the parasite infecting a host. The authors examined the feeding and defecation behaviors of infected and uninfected specimens of *T. dimidiata*, one of the three most epidemiologically important vectors of *Trypanosoma cruzi* to human populations in Mexico.

They found notable differences in the time required to detect the host in individuals infected with *T. dimidiata* (minor) compared to uninfected individuals. Infected individuals bit the host more times and defecated in less than 10 minutes compared to uninfected individuals. More infected individuals defecated within 10 minutes of eating than uninfected individuals. In contrast, similar percentages of both infected and uninfected triatomines defecated during feeding. A similar pattern was observed in feeding times.

I believe this manuscript provides interesting data to unravel the mystery of the influence of *T. cruzi* on the behavior of each triatomine species. Therefore, I believe it deserves to be published in PLoS, but before that happens, some basic questions need to be resolved.

**Lines 85-88**. “The probability of the parasites contained into the feces coming into contact with and infecting a host depends on several parameters of these behaviors, such as feeding duration, number of bites during feeding, and the timing and number of fecal drops. These define an efficient *T. cruzi*  vector and can significantly impact the epidemiology of Chagas disease [3].” The distance from the host of first defecation post feeding likely influences also the chances of infection. Please include that information. ARTÍCULO DE BENJAMÍN 2025.

**Lines 89-93**. “Studies on various triatomine species have shown that those exhibiting a higher number of bites, defecating during or shortly after blood feeding, and engaging in prolonged feeding behavior increase the likelihood of *T. cruzi* transmission, thereby enhancing their vectorial capacity [3–7]. The authors cite two articles on *T. infestans*, two on *T. sordida*, and one on *T. carrioni*. Consider diversifying the species to cite and including some Mexican species, such as the one in their study. These studies are abundant and easily accessible.

**Lines 102-115**. The authors mention the results on various parameters from different authors for various triatomine species, highlighting the differences in favor of infected triatomines compared to uninfected ones. However, it is necessary to include those data (as the authors did in later lines), but even more important is to include those authors' conclusions based on those differences, because in some of them the recorded differences do not convert an ineffective vector into an effective one.

**Lines 117-122**. Studies in which no differences were found between the groups studied should be explained in more detail, similar to those in which differences were observed.

The Introduction would benefit if the authors read section 3.3 (Effects of *T. cruzi* on triatomines) of the chapter:

Guarneri A. A. and G. A. Schaub. 2021. Interaction of triatomines with their bacterial microbiota and trypanosomes. (p. 355-363). In: A. Guarneri and M. Lorenzo (Eds.) *Triatominae.* *The Biology of Chagas Disease Vectors*. Springer.

**Line 123**. “*Triatoma dimidiata* (Latreille)”. Please indicate whether your population belongs to *Triatoma dimidiata sensu lato* or *sensu stricto*. Remember to use this name throughout your manuscript.

**Lines 124-126** “Like other triatomines, it displays diverse behaviors, including, among others, aggregation, alarm responses, defensive actions, host-seeking, feeding, defecation, and mating behaviors”. It is inappropriate to support all this information with a limited reference. Dr. Zeledón's studies should be included, as should those related to *T. dimidiata* from Mexico, including some from the UADY group.

**Line 342**. Modify *Triatoma* ***sórdida***
