# Supplementary material for: Dangerous appetite: The impact of Trypanosoma cruzi infection on the feeding and defecation behaviors of Triatoma dimidiata sensu lato (Latreille, 1811)
Source: PLoS Negl Trop Dis. 2025 Dec 26;19(12):e0013872. doi: 10.1371/journal.pntd.0013872 (PMC12758822; doi:10.1371/journal.pntd.0013872)
Supplement: Attachment — Submitted filename: Response to Reviewers.docx [file pntd.0013872.s004.docx]

**Reviewers' comments:**

Reviewer #1: This is a well structured and defined manuscript looking at the behaviors patters of infected T. dimidiate Vs non-infected. Authors should stated in the meteorology what was the source of feeding and how the host was managed. (is the¡ere a bioethical permission for this?).

**R. We thank Reviewer #1 for this comment. Information regarding bioethical permission has been added (L: 258-260 in the version with track changes). The source of insect feeding was already provided in the original submission in the Materials and Methods section (pigeons, L: 160–161; mice, L: 181 in the version with track changes).**

Reviewer #2: 1. The manuscript does not provide essential information regarding the origin and maintenance of the T. dimidiata colony used in the experiments. Given that T. dimidiata is a species complex with significant genetic and behavioral variation across its geographic distribution, it is critical to specify the collection site, the estimated number of laboratory generations, and the approximate number of founding individuals used to establish the colony. These factors may directly influence the behavioral traits observed and are important for evaluating the ecological relevance of the results.

**R. We thank reviewer #2 for this observation. We have now added the requested information regarding collection sites, number of generations, and the genetics of founding individuals of the colony used in this study in the Materials and Methods section: Insects (L:162-169 in the version with track changes). We also added information regarding the *T. dimidiata* species complex in the Introduction (L: 131-135 in the version with track changes) to enhance coherence throughout the manuscript.**

2. Although the authors acknowledge the limited sample size (n = 20), key methodological details concerning the infection protocol are missing. It is not specified whether infected insects were fed on the same or on different mice, whether host parasitemia was measured or standardized, or whether infection intensity in the insects was quantified. These omissions, particularly in conjunction with the small sample size, may introduce variability and potential bias into the behavioral outcomes. The sample size should be treated as a factor limiting the strength of interpretation, rather than justified by precedent.

**R. We acknowledge the reviewer's concern that such omissions, combined with the small sample size, could potentially introduce variability or bias in behavioral outcomes. Consequently, we now specify the details requested by the reviewer: L: 180-182 in the version with track changes (feeding and host parasitemia) and L: 187-191 in the version with track changes (parasite load in the insects). We also now discuss these limitations (L: 363-376 in the version with track changes). Notably, however, as parasite load assessed in the insects ranged from 1.5 to 2.5 x 10^6^ parasites mL^−1^ of feces, the range observed here, remaining within the same order of magnitude, is unlikely to have biased our results (L:370-376 in the version with track changes).**

3. The starvation period prior to the behavioral assays ranged from 10 to 15 days. This 5-day variation is biologically meaningful for triatomines—especially in nymphal stages—as hunger level can significantly influence host-seeking and feeding behavior. In combination with the small sample size, this variation could affect the consistency of the behavioral measurements. While it may not invalidate the results, it should be acknowledged as a potential limitation in the discussion.

**R.** **We acknowledge the reviewer's concern that the starvation period, ranging from 10 to 15 days before the behavioral assays, combined with the relatively small sample size, could have introduced some variability in host-seeking and feeding behavior. We now discuss this in the limitations in the Discussion section (L: 376-385 in the version with track changes). Notably, however, previous studies indicate that starvation durations within the range of 10–15 days have no significant impact on the feeding responses of triatomines (L: 381-385 in the version with track changes).**

Reviewer #3: The objectives were achieved and corresponded to an adequate hypothesis.
Even though the authors support the low number (20) of individuals examined, I believe that a larger number would have shown more reliable results.
Statistical analysis supported the results.

**R. We thank Reviewer #3 for his/her positive comment.**

**Results:**

Reviewer #1: Results are clear but should briefly describe if possible the degree of ing¡fiction of the vectors......low, medium high? to have a more clear panorama of the situation.

**R. We have added the information regarding the parasitemia range found in the analyzed insects L: 187-191 in the version with track changes.**

Reviewer #2: While clearly presented, may be biased due to methodological limitations outlined in the Methods section.

**R. We now clarified several points in the Materials and Methods section to improve clarity and reproducibility. We have also provided clearer explanations of the statistical tests used and reiterated this information in the Results section to ensure complete transparency throughout the manuscript. In addition, we have addressed specific limitations of the study in the Discussion section (L: 363–385 in the version with track changes).**

Reviewer #3: They found notable differences in the time required to detect the host in individuals infected with T. dimidiata (minor) compared to uninfected individuals. Infected individuals bit the host more times and defecated in less than 10 minutes compared to uninfected individuals. More infected individuals defecated within 10 minutes of eating than uninfected individuals. In contrast, similar percentages of both infected and uninfected triatomines defecated during feeding. A similar pattern was observed in feeding times.
The figures and tables are appropriate and presented in a clear and satisfactory manner.

**R. We thank Reviewer #3 for his/her positive comment.**

**Conclusions:**

Reviewer #1: Conclusion id according the data presented

**R. We thank Reviewer #1 for his/her positive comment.**

Reviewer #2: The manuscript would benefit from a more cautious interpretation of results and a clearer discussion of how these preliminary observations could contribute to broader understanding or public health implications.

**R. We now take a more cautious approach in interpreting the results, which is reflected in the updated Discussion section, and we have largely improved the section addressing the study’s limitations (L: 363-385 in the version with track changes).**

Reviewer #3: The data supported the conclusions reached. The authors clearly described the limitations of their work.
Public health could benefit from a better understanding of the behavior of each triatomine species.

**R. We thank Reviewer #3 for his/her positive comment.**

**Editorial and Data Presentation Modifications?**

Reviewer #1: Probable the inclusion of pictures of fedding bugs will give a more visual impact of the results.

**R. We thank Reviewer #1 for this suggestion. We have included a figure illustrating the key steps of the experimental procedures (Figure 1).**

Reviewer #2: (No Response)

Reviewer #3: Lines 85-88. “The probability of the parasites contained into the feces coming into contact with and infecting a host depends on several parameters of these behaviors, such as feeding duration, number of bites during feeding, and the timing and number of fecal drops. These define an efficient T. cruzi vector and can significantly impact the epidemiology of Chagas disease [3].” The distance from the host of first defecation post feeding likely influences also the chances of infection. Please include that information.
Nogueda-Torres B. et al. (2025). Period of time and movement distances between feeding and postfeeding defecation in Triatoma pallidipennis (Heteroptera: Reduviidae). Acta Trop. 263:107563. doi: 10.1016/j.actatropica.2025.107563.

**R. We thank the reviewer for this suggestion and have added the information (L: 95 in the version with track changes), and two references have been added, Nogueda-Torres et al., 2022, 2025.**

Lines 89-93. “Studies on various triatomine species have shown that those exhibiting a higher number of bites, defecating during or shortly after blood feeding, and engaging in prolonged feeding behavior increase the likelihood of T. cruzi transmission, thereby enhancing their vectorial capacity [3–7]. The authors cite two articles on T. infestans, two on T. sordida, and one on T. carrioni. Consider diversifying the species to cite and including some Mexican species, such as the one in their study. These studies are abundant and easily accessible.

**R. We thank the reviewer for this suggestion and have added two studies on Mexican triatomine species addressing feeding and defecation patterns (Nogueda-Torres et al., 2022, 2025, L: 97 in the version with track changes).**

Lines 102-115. The authors mention the results on various parameters from different authors for various triatomine species, highlighting the differences in favor of infected triatomines compared to uninfected ones. However, it is necessary to include those data (as the authors did in later lines), but even more important is to include those authors' conclusions based on those differences, because in some of them the recorded differences do not convert an ineffective vector into an effective one.

**R. We thank the reviewer for the suggestion. In this section, we do not make any inference about the effectiveness of the vectors presented; we only present, factually, the data reported between infected and uninfected vectors in the cited articles.**

Lines 117-122. Studies in which no differences were found between the groups studied should be explained in more detail, similar to those in which differences were observed.
The Introduction would benefit if the authors read section 3.3 (Effects of T. cruzi on triatomines) of the chapter: Guarneri A. A. and G. A. Schaub. 2021. Interaction of triatomines with their bacterial microbiota and trypanosomes. (p. 355-363). In: A. Guarneri and M. Lorenzo (Eds.) Triatominae. The Biology of Chagas Disease Vectors. Springer.

**R. We thank the reviewer for this suggestion. We have expanded this section, as suggested (L:122-127 in the version with track changes).**

Line 123. “Triatoma dimidiata (Latreille)”. Please indicate whether your population belongs to Triatoma dimidiata sensu lato or sensu stricto. Remember to use this name throughout your manuscript.
**R. We appreciate the suggestion. The information has been added to the Introduction and Materials and Methods sections (L. 131–135 and 162–169 in the version with track changes). Additionally, we now specify that in this study, *Triatoma dimidiata* should be interpreted as referring to *T. dimidiata* sensu lato (L. 168–169 in the version with track changes). We also added “sensu lato” in key parts of the manuscript, including the title, to reinforce this clarification and enhance coherence throughout the manuscript.**

Lines 124-126 “Like other triatomines, it displays diverse behaviors, including, among others, aggregation, alarm responses, defensive actions, host-seeking, feeding, defecation, and mating behaviors”. It is inappropriate to support all this information with a limited reference. Dr. Zeledón's studies should be included, as should those related to T. dimidiata from Mexico, including some from the UADY group.

**R. We have expanded the references and included the study by Dr. Zeledón as well as research specifically focused on *T. dimidiata* from Mexico, including works conducted by our group (L:135-138 in the version with track changes).**

Line 342. Modify Triatoma sórdida

**R. Done (L: 444 in the version with track changes).**

**Summary and General Comments:**

Use this section to provide overall comments, discuss strengths/weaknesses of the study, novelty, significance, general execution and scholarship. You may also include additional comments for the author, including concerns about dual publication, research ethics, or publication ethics. If requesting major revision, please articulate the new experiments that are needed.

Reviewer #1: In the discussion section just to mention that the degree I¡of infection of vectors was not assessed and this may have some effects of the results within the experimental group

**R: We now added information about the range of parasite load measured in the insects (L: 187-191 in the version with track changes). In addition, we added a part in the Discussion section regarding the range of parasite loads (L: 370-376 in the version with track changes).**

Reviewer #2: This manuscript examines the influence of Trypanosoma cruzi infection on the feeding and defecation behaviors of Triatoma dimidiata, an important vector of Chagas disease. While the experimental approach is valid and the topic relevant, the study presents important limitations. In particular, missing methodological details and the restricted scope of the experimental design reduce the interpretability and generalizability of the findings.

**R. We have added further details to the Materials and Methods section to enhance reproducibility and ensure consistency with the Results. In addition, we have addressed and explicitly discussed potential sources of bias (see Lines 363–385 in the version with track changes). We have also provided clearer explanations of the statistical tests used and reiterated this information in the Results section to ensure complete transparency throughout the manuscript.**

Reviewer #3: This is an interesting manuscript from the group of young researchers at UADY linked to the French IRPD group of Dr. Waleckx. As the authors state, this is another of several studies that attempt to clarify whether T. cruzi infection sufficiently modifies the behavior of a triatomine species (T. dimidiata in this case) to increase the chances of the parasite infecting a host. The authors examined the feeding and defecation behaviors of infected and uninfected specimens of T. dimidiata, one of the three most epidemiologically important vectors of Trypanosoma cruzi to human populations in Mexico.

I believe this manuscript provides interesting data to unravel the mystery of the influence of T. cruzi on the behavior of each triatomine species. Therefore, I believe it deserves to be published in PLoS, but before that happens, some basic questions need to be resolved.

**R. We thank Reviewer#3 for his/her positive assessment of our study.**
