# Supplementary material for: Dangerous appetite: The impact of Trypanosoma cruzi infection on the feeding and defecation behaviors of Triatoma dimidiata sensu lato (Latreille, 1811)
Source: PLoS Negl Trop Dis. 2025 Dec 26;19(12):e0013872. doi: 10.1371/journal.pntd.0013872 (PMC12758822; doi:10.1371/journal.pntd.0013872)
Supplement: Attachment — Submitted filename: Response_to_Reviewers_auresp_2.docx [file pntd.0013872.s005.docx]

**Reviewers' comments:**

Reviewer's Responses to Questions

**Methods**

Reviewer #1: The methodology is clearly described. Only wondering if authors look for T. Cruzi forms o DNA from the feces of insects after feeding. In Figure 1, was the mice sedated? more information is required.

**R:** We did not perform molecular analyses. The motile, flagellated forms of the parasite were identified through microscopic examination, as described in the Methodology section of the manuscript. We added this information for clarification (Lines 181-182). Additionally, we have added the information in the legend of Figure 1.

Reviewer #2: Since the limitations of the manuscript were acknowledged, I think it is now suitable.

**R:** We thank Reviewer#2 for his/her comment.

Reviewer #3: The objectives are clear and aligned with the hypothesis.
The design is appropriate and leads to the achievement of the objective.

**R:** We thank Reviewer#3 for his/her comment.

**Results**

Reviewer #1: Only wondering if authors look for T. Cruzi forms o DNA from the feces of insects after feeding.

**R:** We did not perform molecular analyses. The motile, flagellated forms of the parasite were identified through microscopic examination, as described in the Methodology section of the manuscript. We added this information for clarification (Lines 181-182).

Reviewer #2: "yes" to all

**R:** We thank Reviewer#2 for his/her comment.

Reviewer #3: The analysis is correct. The results are presented correctly, and the figures and tables help to better understand the study.

**R:** We thank Reviewer#3 for his/her comment.

**Conclusions**


Reviewer #1: Conclusions are clear and concise.

**R:** We thank Reviewer#1 for his/her comment.

Reviewer #2: "yes" to all.

**R:** We thank Reviewer#2 for his/her comment.

Reviewer #3: The data presented support the conclusion, and the limitations of the study are mentioned. The relevance of the study to public health is discussed. The Discussion is useful and clear.

**R:** We thank Reviewer#3 for his/her comment.

**Editorial and Data Presentation Modifications?**

Reviewer #1: Indicate details of figure 1 as stated above

**R:** We have now added the requested information.

Reviewer #2: Accept

Reviewer #3: The minor modifications previously suggested were followed.

**Summary and General Comments**


Reviewer #1: This is an interesting and well written manuscript. Considering changes made as reviewers requested; now it may be considered for publication

**R:** We thank Reviewer#1 for his comment.

Reviewer #2: Since the limitations of the manuscript were acknowledged, I think it is now suitable.

**R:** We thank Reviewer#2 for his/her comment.

Reviewer #3: The minor modifications previously suggested were followed, so I believe this manuscript could be published in its current form.

**R:** We thank Reviewer#3 for his/her comment.
